# Supplementary material for: A simple mortality risk prediction score for viper envenoming in India (VENOMS): A model development and validation study
Source: PLoS Negl Trop Dis. 2022 Feb 22;16(2):e0010183. doi: 10.1371/journal.pntd.0010183 (PMC8896694; doi:10.1371/journal.pntd.0010183)
Supplement: S1 Appendix Text — Table A: Publications screened for variable selection for model development Table B: (Supplementary Appendix 1): Area under the curve (AUC) for Receiver-operating curves (ROC) constructed for continuous predictor variables with mortality (or survival) as the state variable with confidence interval (CI), cut off chosen and sensitivity and specificity at chosen cut-off. Table C: Odds ratio with 95% CI for univariable Binary Logistic Regression (unadjusted) and subsequent multivariable logistic regression with backward elimination strategy (adjusted) to predict mortality as outcome. Table D: Multivariable logistic regression model with backward elimination at step 5 and step 7 Table E: Model summary showing -2 log likelihood, Cox and Snell’s R square, Nagelkerke R Square and Akaike Information criteria (AIC) shown for each step of backward elimination. Fig A: Perfect Internal calibration in derivation cohort (slope of 1, intercept of 0 and an AUC of 0.95). Graph created using pmcalplot in STATA, Stata/IC 16 for Windows. (DOCX) [file pntd.0010183.s002.docx]

**Supplementary Appendix S1**

**Table A:** Publications screened for variable selection for model development.

|  | **Authors (Year of publication)** | **Type of study** | **Variables Considered** | **Comments** |
| --- | --- | --- | --- | --- |
| 1 | Priyamvadha et al 2020 [1]  (Puducherry, India) | Prospective | Mechanical ventilation, hypotension, capillary leak syndrome | Viper bites in adults |
| 2 | Suryanarayana G et al. 2020 [2]  (Puducherry, India) | Retrospective | Bite to ASV time, Presence of torniquet, Walking after bite | Paediatric  population |
| 3 | Gantait et al 2019 [3]  (West Bengal, India) | Prospective | Bite to antivenom time, requirement for Renal replacement therapy |  |
| 4 | Mana K et al 2019 [4]  (West Bengal, India) | Prospective | Bite to ASV time | Primary care multicentre |
| 5 | Kumar S et al. 2018 [5]  (Kerala, India) | Prospective | Capillary leak syndrome,  Albuminuria, neutrophilic leucocytosis, thrombocytopenia, acute kidney injury | Predominantly Russell’s Viper bites |
| 6 | Kyi-Phyu Aye et al. 2018 [6]  (Myanmar) | Prospective | Duration from bite to arrival at the hospital >4 h, white blood cell counts >20 ×10^3^ cells·μL-1 presence of capillary leakage | Predominantly Russell’s Viper bites |
| 7 | Da silva Souza et al 2018 [7]  (Brazil) | Prospective | Age ≥61 years, Indigenous status, lack of antivenom administration, Shock |  |
| 8 | Yu-Hsuan Hsieh ey al 2017 [8]  (Taiwan) | Retrospective | None | Predominantly *Naja naja* bites |
| 9 | Aktar F et al. 2016 [9]  (Iran) | Retrospective | Rural occurrence White blood cell count Aspartate aminotransferase to alanine aminotransferase (AST/ALT) ratio, hypoalbuminemia, hypo-calcemia. | Paediatric  population |
| 10 | Pore et al 2015 [10]  (West Bengal, India) | Retrospective | None | Neurotoxic manifestations were only significant predictor of mortality |
| 9 | Chaudhary et al 2014  (Maharashtra India) [11] | Prospective | Bleeding tendency, mean PT (sec), respiratory failure, shock, mean ASV dose | Neurotoxic and viper bites considered.  (adults) |
| 10 | Sankar et al 2013 [12]  (Puducherry, India) | Prospective | Younger age, walking for >1 km after the bite, haemoglobin ≤10 g/dl at admission | Age < 12 years included |
| 11 | Myo-Khin et al 2012 [13]  (Myanmar) | Prospective | Urine output of <400 ml in the first 24 hours, un-clotted blood, bite-to-injection time of >2 hours |  |
| 12 | Habib et al 2011 [14]  (Nigeria) | Prospective | New CNS symptoms, Shock, Anaemia, Delay in ASV |  |
| 13 | Suchitra N et al. 2008 [15]  (Kerala, India) | Prospective | Capillary leak syndrome, respiratory paralysis, intracerebral bleeding, Bite to ASV time > 6 hours | Predominantly Russell’s Viper bites |
| 14 | Kalantri SP et al 2006 [16]  (Maharashtra India) | Prospective | Vomiting, neurotoxicity and admission serum creatinine | Rural Hospital |

* ASV: Antivenom

**Search Strategy:**

Studies were identified through searches of PubMed for articles published from 1^st^ January 2000 to 28^th^ February 2021, by use of the search terms “snakebite”, “envenoming” and “Russell’s viper” in combination with the term “prognosis”, “mortality”, “score”, “clinical profile”, “prognostic model”, “risk factors”, and “predictors”. Relevant articles were also identified through searches in the authors’ personal files and in Google Scholar. Articles resulting from these searches and relevant references cited in those articles were reviewed. Articles published in English language were only included.

**Potential variables considered:**

1. Need for mechanical ventilation
2. Hypotension
3. Bite to antivenom time >6 hours
4. Capillary leak syndrome
5. Age >65 years
6. Aspartate aminotransferase to alanine aminotransferase (AST/ALT) ratio
7. Hypoalbuminemia
8. Hypo-calcemia
9. Bleeding manifestations
10. Presence of torniquet
11. Walking after bite
12. Haemodialysis requirement
13. Albuminuria
14. Leucocytosis (>20,000/cu mm)
15. Thrombocytopenia
16. Acute kidney injury
17. Haemoglobin < 10 g/dL
18. Vomiting
19. Serum Creatinine
20. Intracranial hemorrhage
21. Anaemia
22. Presence of Shock
23. Urine output of <400 ml in the first 24 hours
24. Bite to antivenom time >2 hours
25. High antivenom dose

**References**

1. Priyamvada PS, Jaswanth C, Zachariah B, Haridasan S, Parameswaran S, Swaminathan RP. Prognosis and long-term outcomes of acute kidney injury due to snake envenomation. Clin Kidney J. 2020;13:564–70.

2. Suryanarayana G, Rameshkumar R, Mahadevan S. Retrospective Hospital-Based Cohort Study on Risk Factors of Poor Outcome in Pediatric Snake Envenomation. J Trop Pediatr. 2020;

3. Gantait K, Patra S, Chowdhury R, Pramanick S. Viper bite and its complications at a tertiary care centre in southern part of West Bengal: A prospective, clinical, socioeconomic and epidemiological study. Natl Med J India. 2019;32:13–6.

4. Mana K, Ghosh R, Gantait K, Saha K, Parua P, Chatterjee U, et al. Incidence and treatment of snakebites in West Bengal, India. Toxicol Rep. 2019;6:239–43.

5. Kumar KS, Narayanan S, Udayabhaskaran V, Thulaseedharan NK. Clinical and epidemiologic profile and predictors of outcome of poisonous snake bites - an analysis of 1,500 cases from a tertiary care center in Malabar, North Kerala, India. Int J Gen Med. 2018;11:209–16.

6. Aye K-P, Thanachartwet V, Soe C, Desakorn V, Chamnanchanunt S, Sahassananda D, et al. Predictive Factors for Death After Snake Envenomation in Myanmar. Wilderness Environ Med. 2018;29:166–75.

7. da Silva Souza A, de Almeida Gonçalves Sachett J, Alcântara JA, Freire M, Alecrim M das GC, Lacerda M, et al. Snakebites as cause of deaths in the Western Brazilian Amazon: Why and who dies? Deaths from snakebites in the Amazon. Toxicon Off J Int Soc Toxinology. 2018;145:15–24.

8. Hsieh Y-H, Hsueh J-H, Liu W-C, Yang K-C, Hsu K-C, Lin C-T, et al. Contributing Factors for Complications and Outcomes in Patients With Snakebite: Experience in a Medical Center in Southern Taiwan. Ann Plast Surg. 2017;78:S32–6.

9. Aktar F, Aktar S, Yolbas I, Tekin R. Evaluation of Risk Factors and Follow-Up Criteria for Severity of Snakebite in Children. Iran J Pediatr. 2016;26:e5212.

10. Pore SM, Ramanand SJ, Patil PT, Gore AD, Pawar MP, Gaidhankar SL, et al. A retrospective study of use of polyvalent anti-snake venom and risk factors for mortality from snake bite in a tertiary care setting. Indian J Pharmacol [Internet]. 2015 [cited 2020 Sep 8];47:270. Available from: https://www.ncbi.nlm.nih.gov/pmc/articles/PMC4450551/

11. Chaudhari TS, Patil TB, Paithankar MM, Gulhane RV, Patil MB. Predictors of mortality in patients of poisonous snake bite: Experience from a tertiary care hospital in Central India. Int J Crit Illn Inj Sci. 2014;4:101–7.

12. Sankar J, Nabeel R, Sankar MJ, Priyambada L, Mahadevan S. Factors affecting outcome in children with snake envenomation: a prospective observational study. Arch Dis Child. 2013;98:596–601.

13. Myo-Khin, Theingi-Nyunt, Nyan-Tun-Oo, Ye-Hla. Prognostic indicators in patients with snakebite: analysis of two-year data from a township hospital in central Myanmar. WHO South-East Asia J Public Health. 2012;1:144–50.

14. Habib AG, Abubakar SB. Factors affecting snakebite mortality in north-eastern Nigeria. Int Health. 2011;3:50–5.

15. Suchithra N, Pappachan JM, Sujathan P. Snakebite envenoming in Kerala, South India: clinical profile and factors involved in adverse outcomes. Emerg Med J EMJ. 2008;25:200–4.

16. Kalantri S, Singh A, Joshi R, Malamba S, Ho C, Ezoua J, et al. Clinical predictors of in-hospital mortality in patients with snake bite: a retrospective study from a rural hospital in central India. Trop Med Int Health TM IH. 2006;11:22–30.

**Table B** (Supplementary Appendix 1): Area under the curve (AUC) for Receiver-operating curves (ROC) constructed for continuous predictor variables with mortality (or survival) as the state variable with confidence interval (CI), cut off chosen and sensitivity and specificity at chosen cut-off.

| **S/no** | **Variable** | **AUC** | **95% CI** | ***p*-value** | **Chosen cut-off value** | **Sensitivity (%)** | **Specificity (%)** |
| --- | --- | --- | --- | --- | --- | --- | --- |
| 1 | Bite to ASV time (h) | 0.602 | 0.52-0.68 | 0.019 | 6.5 h | 56.1 | 59.2 |
| 2 | SBP at admission  (mm Hg) | 0.752 | 0.67-0.80 | < 0.0001 | 100 mm Hg | 78.5 | 64.9 |
| 3 | Total ASV dose  (ml) | 0.65 | 0.57-0.73 | < 0.0001 | 305 ml | 63.2 | 52.9 |
| 4 | Haemoglobin | 0.70 | 0.62-0.78 | < 0.0001 | 10.1g/dL | 76.2 | 50.9 |
| 5 | Platelet count (cu mm) | 0.74 | 0.68-0.81 | < 0.0001 | 26,000 | 76.7 | 56.1 |
| 6 | INR | 0.70 | 0.62-0.78 | < 0.0001 | 1.62 | 66.7 | 70.7 |
| 7 | Serum Creatinine at admission | 0.66 | 0.58-0.73 | < 0.0001 | 3.0 mg/dL | 76.9 | 54.3 |
| 8 | Urine output one-hour post admission | 0.87 | 0.81-0.91 | < 0.0001 | 20 ml | 67.2 | 90.1 |
| 9 | Age | 0.45 | 0.45-0.62 | 0.340 | 50 years | 31.4 | 66 |

ASV: Antivenom, SBP: Systolic Blood Pressure

**Table C:** Odds ratio with 95% CI for univariable Binary Logistic Regression (unadjusted) and subsequent multivariable logistic regression with backward elimination strategy (adjusted) to predict mortality as outcome.

|  | **Risk Factor/Variable** | **Unadjusted Odds Ratio (OR)** | **P value (95% Confidence Interval)** | **Adjusted**  **Odds Ratio* (OR)**  **(Step eliminated)** | **P value (95% Confidence Interval)** |
| --- | --- | --- | --- | --- | --- |
| 1 | Age | 0.99 | 0.37  (0.97-1.01) | 0.69  (Step 2) | 0.51  (0.21-2.12) |
| 2 | Female Gender | 2.12 | 0.15  (1.15-3.91) | 2.47***** | 0.084  (0.89-6.87) |
| 3 | Presence of CLS | 19.6 | <0.0001  (9.45-40.80) | 8.83***** | <0.0001  (3.33-23.42) |
| 4 | Overt Bleeding | 23.4 | <0.0001  (7.07-77.5) | 17.26***** | < 0.0001  (3.84-77.57) |
| 5 | Bite to ASV time > 6.5h | 1.85 | 0.04  (1.02-3.37) | 1.934***** | 0.109  (0.74-5.08) |
| 6 | SBP at admission <100 mm Hg | 6.76 | <0.0001  (3.55-12.89) | 6.59***** | < 0.0001  (2.44-17.77) |
| 7 | Hb <10g/dL | 3.46 | <0.0001  (1.85-6.49) | 2.23***** | 0.108  (0.84-6.10) |
| 8 | Platelet <26,000/cu mm | 3.38 | <0.0001  (1.81-6.30) | 0.75  (Step 3) | 0.598  (0.25-2.16) |
| 9 | Serum Creatinine >3.0 mg/dL | 3.95 | <0.0001  (1.95-8.01) | 1.22  (Step 2) | 0.78  (0.30-4.98) |
| 10 | Urine Albumin positive (by dipstick) | 2.44 | 0.024  (1.13- 5.30) | 1.22  (Step 3) | 0.76  (0.34- 4.34) |
| 11 | Severity of local envenoming (four grades) ^#^ | 2.2 | 0.31  (0.46-10.53) | 4.59  (Step 2) | 0.97  0.00-NA |
| 12 | Urine output < 20 ml/h on day 1 of admission | 17.27 | <0.0001  (7.02-42.39) | 8.78***** | < 0.0001  (2.84-27.15) |

ASV: Antivenom, CLS: Capillary leak syndrome, Hb: Haemoglobin, SBP: Systolic Blood Pressure. # None: No swelling, Mild: Swelling at bite site. Moderate: Swelling crossing one joint proximal to bite site or presence of significant local lymph-nodes, Severe: swelling involving more than one joint proximal to bite site/presence of necrosis/compartment syndrome/facial or trunk bite. Urine output was measured over 24 hours of admission and later converted to ml/hour.

**Table D: Multivariable logistic regression model with backward elimination at step 5 and step 7**

| **Step 5** | | | | | | | | |
| --- | --- | --- | --- | --- | --- | --- | --- | --- |
| **Parameter** | **β** | **SE** | **Wald** | **df** | **P value** | **Exp (B)** | **95% CI** | |
| Gender | 0.903 | 0.522 | 2.990 | 1 | 0.084 | 2.467 | 0.89 | 6.87 |
| CLS | 2.178 | 0.498 | 19.169 | 1 | 0.000 | 8.833 | 3.33 | 23.44 |
| B to ASV | 0.660 | 0.493 | 1.789 | 1 | 0.109 | 1.934 | 0.74 | 5.08 |
| Bleeding | 2.848 | 0.767 | 13.793 | 1 | 0.000 | 17.256 | 3.84 | 77.57 |
| Hb | 0.806 | 0.503 | 2.568 | 1 | 0.108 | 2.238 | 0.84 | 6.10 |
| U/O 20 | 2.173 | 0.576 | 14.237 | 1 | 0.000 | 8.783 | 2.84 | 27.15 |
| SBP < 100 | 1.888 | 0.506 | 13.875 | 1 | 0.000 | 6.589 | 2.44 | 17.77 |
| Constant | -7.276 | 1.105 | 43.383 | 1 | 0.000 | 0.001 | --- | --- |
| **Step 7** | | | | | | | | |
| **Parameter** | **β** | **SE** | **Wald** | **df** | **P value** | **Exp (B)** | **95% CI** | |
| Gender | 0.853 | 0.502 | 2.890 | 1 | 0.089 | 2.347 | 0.88 | 6.28 |
| CLS | 2.230 | 0.487 | 20.986 | 1 | 0.000 | 9.298 | 3.50 | 24.14 |
| Bleeding | 2.632 | 0.719 | 13.397 | 1 | 0.000 | 13.904 | 3.41 | 56.92 |
| U/O 20 | 2.253 | 0.567 | 15.798 | 1 | 0.000 | 9.514 | 3.13 | 28.80 |
| SBP < 100 | 1.986 | 0.499 | 15.842 | 1 | 0.000 | 7.287 | 2.74 | 19.38 |
| Constant | -6.583 | 0.962 | 46.782 | 1 | 0.000 | 0.001 | --- | --- |

B to ASV: Bite to antivenom time >6.5 hours, CLS: Capillary leak syndrome, Hb: Haemoglobin < 10g/dL, SBP <100: Systolic Blood Pressure <100 mm Hg, U/O 20: Urine output < 20 ml/h on day 1 of admission. NOTE: Important clinically relevant parameters Bite to ASV time is eliminated by step 7.

**Table E: Model summary showing -2 log likelihood, Cox and Snell’s R square, Nagelkerke R Square and Akaike Information criteria (AIC) shown for each step of backward elimination.**

| **Step of Backward Elimination** | **- 2 log likelihood** | **df** | **Cox and Snell’s R square** | **Nagelkerke**  **R Square** | **AIC** |
| --- | --- | --- | --- | --- | --- |
| Step 1 | 114.278 | 13 | 0.461 | 0.697 | 140.278 |
| Step 2 | 114.562 | 10 | 0.460 | 0.696 | 137.562 |
| Step 3 | 114.638 | 9 | 0.460 | 0.696 | 132.638 |
| Step 4 | 114.319 | 8 | 0.460 | 0.695 | 130.919 |
| **Step 5** | **115.297** | **7** | **0.459** | **0.694** | **129.297** |
| Step 6 | 117.118 | 6 | 0.455 | 0.688 | 129.118 |
| Step 7 | 119.317 | 5 | 0.450 | 0.680 | 129.317 |

**NOTE:** AIC best is in step 6, but step 5 was chosen to retain clinically relevant parameters.

**Fig A: Perfect Internal calibration in derivation cohort (slope of 1, intercept of 0 and an AUC of 0.95).**


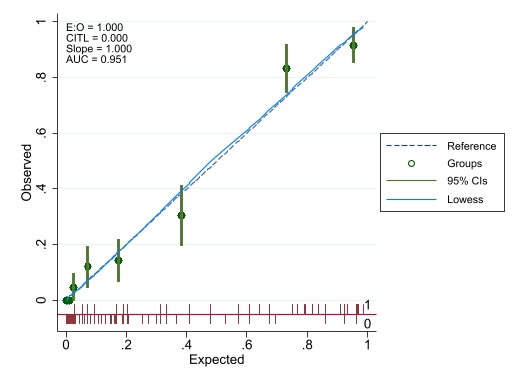


Graph created using pmcalplot
