# Supplementary material for: A simple mortality risk prediction score for viper envenoming in India (VENOMS): A model development and validation study
Source: PLoS Negl Trop Dis. 2022 Feb 22;16(2):e0010183. doi: 10.1371/journal.pntd.0010183 (PMC8896694; doi:10.1371/journal.pntd.0010183)
Supplement: S1 Data — (PDF) [file pntd.0010183.s003.pdf]

slnum	Age	Gender	Duration_s	Outcome	cls0002	AKI008	Bitecode_6	Bitetoneed
1	22	F	4	DIS	0	0	0	2
2	27	F	4	DIS	0	0	0	3
3	50	M	7	DIS	0	1	0	5
4	27	F	13	DIS	0	1	0	2
5	60	M	7	DIS	0	0	0	3
6	35	M	10	DIS	0	1	0	3
7	30	M	7	DIS	0	1	0	5
8	50	F	20	DIS	0	1	1	12
9	25	M	8	DIS	1	1	1	8
10	35	F	8	DIS	0	0	1	24
11	55	M	5	DIS	0	0	0	4
12	17	M	5	DIS	0	0	0	2
13	40	F	10	DIS	0	0	0	5
14	50	M	11	DIS	0	1	0	1
15	22	M	7	DIS	0	0	0	6
16	19	M	6	DIS	0	0	1	24
17	23	M	5	DIS	0	1	1	8
18	26	M	4	DIS	0	0	1	24
19	40	M	6	DIS	0	0	1	10
20	70	M	6	DIS	0	1	1	20
21	36	M	3	DIS	0	0	1	24
22	54	M	6	DIS	0	0	1	10
23	45	M	8	DIS	0	0	0	4
24	42	M	6	DIS	0	0	1	9
25	34	M	5	DIS	0	0	1	12
26	28	M	20	DIS	0	0	0	5
27	21	M	6	DIS	0	0	1	24
28	28	M	6	DIS	0	1	1	18
29	41	M	5	DIS	1	1	1	12
30	21	M	5	DIS	0	1	0	5
31	56	M	6	DIS	0	1	0	2
32	35	F	5	DIS	0	0	0	5
33	38	F	6	DIS	0	0	0	3
34	14	F	4	DIS	0	0	1	24
35	32	F	8	DIS	0	0	0	5
36	42	F	4	DIS	0	0	0	4
37	50	F	5	DIS	1	0	0	1
38	76	M	4	DIS	0	0	1	15
39	28	M	19	DIS	0	0	1	24
40	25	M	4	DIS	0	1	0	1
41	53	M	10	DIS	0	1	1	24
42	59	M	8	DIS	0	1	1	8
43	64	M	13	DIS	0	1	1	12
44	17	M	5	DIS	0	1	1	24
45	72	F	22	DIS	0	1	1	12
46	28	M	4	DIS	0	0	0	2
47	56	M	6	DIS	0	0	0	6
48	45	F	16	DIS	0	1	0	5
49	28	F	3	DIS	0	0	1	48

50	50	M	4	DIS	0	0	0	5
51	37	M	4	EXP	1	1	1	24
52	26	M	7	EXP	0	1	0	2
53	30	F	4	EXP	1	1	1	24
54	21	M	9	EXP	1	1	0	2
55	35	F	50	EXP	1	1	0	1.5
56	30	M	3	EXP	0	1	0	5
57	50	M	3	EXP	1	1	1	8
58	73	M	3	EXP	0	1	0	1
59	50	F	6	EXP	0	1	0	2
60	22	M	7	EXP	0	1	0	2
61	36	F	3	EXP	1	1	1	12
62	55	M	1	EXP	0	1	0	3
63	50	F	1	EXP	1	1	0	2
64	75	F	3	EXP	0	1	0	3
65	29	F	1	EXP	1	1	0	5
66	45	F	5	DIS	0	0	1	11
67	19	M	4	EXP	1	0	0	4
68	34	M	7	DIS	0	1	1	11
69	24	M	11	DIS	0	1	0	2
70	62	M	6	DIS	1	0	0	4
71	29	M	7	DIS	0	1	0	2
72	65	M	7	DIS	0	1	1	8
73	23	M	8	DIS	0	1	1	9
74	15	M	8	DIS	1	1	0	1
75	21	M	5	DIS	0	0	1	24
76	30	M	3	DIS	0	0	0	2
77	45	M	10	DIS	0	1	1	48
78	53	M	14	DIS	0	1	0	4
79	32	M	5	DIS	0	0	1	20
80	40	M	4	DIS	0	0	1	96
81	60	M	5	DIS	0	0	0	4
82	33	M	5	DIS	0	0	1	8
83	46	M	5	DIS	0	0	0	5
84	58	M	5	DIS	0	0	1	7
85	45	M	10	DIS	0	1	1	7
86	65	M	5	DIS	0	1	1	8
87	39	M	53	DIS	1	1	0	2
88	37	M	7	DIS	0	1	1	26
89	28	M	13	DIS	0	1	1	22
90	30	M	6	DIS	0	1	1	10
91	25	M	9	DIS	0	1	0	6
92	56	F	5	DIS	0	0	1	24
93	52	M	13	DIS	0	1	1	48
94	40	F	7	DIS	0	0	1	18
95	47	F	5	DIS	0	1	0	2
96	60	F	8	DIS	0	1	0	6
97	45	F	8	DIS	0	0	0	4
98	28	F	8	DIS	0	0	0	5
99	55	M	14	DIS	0	1	0	5

100	50	M	20	DIS	0	0	1	12
101	35	M	9	DIS	0	1	0	2
102	71	M	22	DIS	0	1	0	1
103	56	F	8	DIS	0	1	0	5
104	55	M	20	DIS	0	1	1	18
105	40	M	6	DIS	0	0	1	8
106	20	M	8	DIS	1	1	0	4
107	22	M	4	DIS	0	0	0	2
108	50	M	16	DIS	0	1	0	6
109	37	M	7	DIS	0	1	0	2
110	58	M	5	DIS	0	1	0	6
111	55	M	11	DIS	1	1	0	1
112	49	F	7	DIS	0	0	0	1
113	55	M	8	DIS	0	0	0	2
114	43	M	13	DIS	0	0	0	2
115	42	M	6	DIS	0	0	1	14
116	40	M	15	DIS	1	1	0	4
117	60	M	6	DIS	0	0	0	1
118	23	M	4	DIS	0	0	0	6
119	30	M	3	DIS	0	0	0	1
120	16	M	5	DIS	0	0	0	1
121	38	M	120	EXP	1	1	0	6
122	17	M	12	DIS	1	1	0	1
123	60	M	8	DIS	0	0	0	4
124	38	M	11	DIS	0	1	0	3
125	42	F	15	DIS	1	1	0	4
126	22	F	7	DIS	0	1	0	3
127	34	F	13	DIS	0	0	1	8
128	60	F	13	DIS	0	1	1	48
129	55	M	1	EXP	1	1	0	5
130	35	F	3	DIS	0	0	1	12
131	42	F	3	DIS	0	0	0	2
132	19	F	6	DIS	0	0	0	5
133	41	M	7	DIS	0	1	0	3
134	13	M	9	DIS	0	1	1	72
135	26	M	1	EXP	0	0	0	4
136	45	F	24	DIS	0	1	0	6
137	40	M	10	DIS	0	1	0	5
138	45	F	17	DIS	0	1	0	3
139	40	F	7	DIS	0	1	0	6
140	16	M	10	DIS	1	1	1	12
141	40	F	15	DIS	0	1	1	10
142	39	M	4	DIS	0	1	1	15
143	25	M	5	DIS	0	0	1	9
144	42	M	19	DIS	0	1	1	48
145	64	M	4	DIS	0	0	0	5
146	46	M	3	EXP	0	1	0	5
147	18	F	1	EXP	1	1	0	2
148	13	F	1	EXP	1	1	1	12
149	40	M	1	EXP	0	1	1	18

150	30	F	1	EXP	1	1	1	9
151	25	M	4	EXP	1	1	1	72
152	49	F	7	DIS	0	0	0	3
153	39	M	3	DIS	0	0	0	2
154	14	F	4	DIS	0	0	0	1
155	45	M	2	DIS	0	0	0	5
156	45	M	7	DIS	0	0	0	5
157	50	F	2	EXP	0	0	1	12
158	60	F	3	EXP	0	1	1	24
159	35	M	4	EXP	1	1	0	3
160	30	M	1	EXP	1	1	1	15
161	16	M	1	EXP	0	0	1	10
162	60	M	7	EXP	0	1	1	48
163	45	F	1	EXP	1	1	1	18
164	50	F	5	EXP	1	1	1	72
165	50	M	2	EXP	0	1	0	6
166	16	F	1	EXP	1	1	1	9
167	43	M	1	EXP	1	1	1	15
168	52	F	1	EXP	0	1	1	48
169	20	F	19	EXP	1	1	1	12
170	45	F	5	EXP	1	1	1	12
171	55	M	17	EXP	1	1	1	15
172	22	M	1	EXP	1	1	1	12
173	50	F	1	EXP	1	0	1	24
174	45	M	2	EXP	1	0	1	7
175	35	F	1	EXP	1	1	1	12
176	38	F	2	EXP	1	1	0	5
177	17	F	4	EXP	1	1	1	12
178	50	F	4	EXP	1	1	1	24
179	14	M	1	EXP	1	1	0	5
180	22	M	9	EXP	1	1	0	6
181	31	M	39	EXP	1	1	0	5
182	65	M	2	EXP	1	1	1	96
183	54	M	3	EXP	1	1	1	9
184	65	F	11	DIS	0	1	1	48
185	40	M	10	DIS	0	1	1	15
186	60	F	6	DIS	0	1	0	1
187	45	M	6	DIS	0	1	0	6
188	40	F	10	DIS	0	1	0	3
189	40	M	8	DIS	0	1	1	12
190	55	M	4	DIS	0	0	0	2
191	70	F	8	DIS	0	1	1	48
192	58	M	4	DIS	0	1	0	4
193	29	M	7	DIS	0	1	0	5
194	30	M	1	EXP	0	1	1	8
195	28	F	1	EXP	1	1	1	20
196	37	M	20	EXP	0	1	0	6
197	35	M	29	DIS	0	1	1	48
198	28	F	3	EXP	1	1	1	12
199	68	F	3	EXP	1	1	0	1

200	19	M	4	DIS	0	0	0	1
201	54	F	1	DIS	0	0	0	1
202	35	F	16	DIS	0	1	1	48
203	70	F	8	DIS	0	0	1	10
204	19	F	9	DIS	0	1	1	24
205	45	M	1	DIS	0	0	0	5
206	25	M	19	DIS	0	0	1	8
207	16	M	7	DIS	1	1	0	1
208	35	M	6	DIS	0	0	1	48
209	60	M	4	DIS	1	1	1	7
210	35	M	4	DIS	1	1	1	15
211	39	M	2	DIS	0	0	0	1
212	19	M	9	DIS	0	1	1	24
213	60	M	6	DIS	0	1	0	1
214	60	M	18	DIS	0	1	0	6
215	16	M	8	DIS	0	1	0	5
216	40	F	6	DIS	0	1	0	1
217	35	M	5	DIS	0	1	1	48
218	55	M	6	DIS	0	1	0	1
219	21	M	5	DIS	0	1	1	24
220	28	M	7	DIS	0	1	0	3
221	19	F	8	DIS	0	1	0	3
222	33	M	15	DIS	0	1	0	2
223	18	M	18	DIS	1	1	1	24
224	40	M	8	DIS	1	1	0	1
225	45	M	23	DIS	0	1	0	2
226	35	F	12	DIS	1	1	0	1
227	71	F	19	DIS	0	1	0	1
228	40	F	7	DIS	0	1	0	3
229	62	M	12	DIS	0	1	0	3
230	45	F	20	DIS	0	1	0	1
231	40	M	6	DIS	1	1	0	1
232	50	M	7	DIS	0	1	0	3
233	17	M	14	DIS	0	1	1	12
234	50	F	5	DIS	0	1	1	8
235	67	M	7	DIS	0	1	0	5
236	27	M	13	DIS	0	1	1	12
237	17	F	3	DIS	0	0	0	1
238	55	F	6	DIS	0	1	0	1
239	45	M	1	DIS	0	0	0	1
240	30	M	2	DIS	0	0	0	1
241	55	M	2	DIS	0	0	1	8
242	55	M	14	DIS	0	1	0	5
243	60	M	8	DIS	0	1	1	72
244	25	M	4	DIS	0	0	1	18
245	57	M	4	DIS	0	0	1	24
246	55	M	7	DIS	0	0	0	1
247	28	M	5	DIS	0	0	0	1
248	41	M	16	DIS	0	1	0	1

cell_severit	SBP_cat	SBPadmin	Hb_cat	U0_cat	UOadmin	Bleedin_ca	CLS	creat_a
3	1	100	0	0	1500	0	N	0.6
3	0	110	0	0	1500	0	N	1.2
3	0	130	0	0	2000	0	N	3.5
3	0	150	1	0	1600	0	N	6.7
2	0	120	0	0	2000	0	N	1.3
3	0	144	0	1	400	0	N	9
1	1	90	0	0	2000	1	N	4.7
3	0	150	1	1	50	1	N	7.1
2	0	110	0	0	1200	0	Y	1.2
3	0	144	1	0	2500	0	N	4
2	0	110	0	0	1800	0	N	1
2	0	120	1	0	1500	0	N	1.6
2	1	100	0	0	500	0	N	4.6
2	0	130	0	0	1200	0	N	1
1	0	130	0	0	2000	1	N	0.6
3	0	116	0	0	500	0	N	32
2	1	80	0	0	1200	0	N	1
3	0	110	0	0	1250	1	N	0.6
2	0	130	0	0	1200	0	N	0.7
3	0	140	0	0	960	0	N	1.8
2	1	80	1	0	1200	0	N	0.6
3	0	120	0	0	1350	1	N	1.3
3	0	160	0	0	900	1	N	1
3	0	130	0	0	650	1	N	2.3
3	1	100	0	0	950	1	N	1.2
3	0	110	0	0	500	1	N	#NULL!
2	1	100	0	0	850	0	N	1.2
3	0	138	1	1	200	0	N	11
2	1	100	0	1	60	1	Y	1
1	0	110	0	0	600	0	N	4
3	0	110	0	0	960	0	N	1.1
3	0	110	1	0	1700	0	N	0.77
2	0	120	1	0	1600	1	N	1.9
3	0	120	1	0	1500	0	N	0.8
3	0	130	0	0	1650	0	N	0.8
3	0	110	0	0	1800	1	N	0.9
1	0	120	0	0	2600	1	Y	0.8
3	0	110	0	0	500	0	N	1.6
3	0	130	0	1	200	0	N	#NULL!
1	0	110	0	0	1200	0	N	0.6
2	0	110	1	1	40	1	N	12.7
1	0	130	0	1	450	0	N	2.8
3	0	110	1	1	400	1	N	6.1
3	0	140	0	0	1250	1	N	3.4
3	0	130	0	0	600	1	N	5.1
1	0	120	0	0	1500	0	N	1.2
1	0	102	0	0	1200	1	N	1.7
2	0	130	0	1	50	0	N	3.8
2	0	120	1	0	1320	0	N	1.1

1	1	96	0	0	1200	0	N	0.9
3	0	130	1	0	500	1	Y	2.6
3	0	110	1	1	150	1	N	7.1
3	0	108	1	1	40	1	Y	4
3	0	130	0	1	450	1	Y	8.5
3	0	110	1	1	150	1	Y	12.6
3	1	90	1	1	200	1	Y	3.2
2	0	130	0	1	400	1	Y	4.5
3	1	90	1	1	250	1	N	6.1
1	0	120	0	0	800	1	Y	0.8
3	0	140	1	1	230	1	Y	7.4
3	1	100	0	1	40	1	Y	5.3
2	1	40	1	1	0	1	Y	5
2	0	130	1	1	0	1	Y	11.2
3	1	84	1	1	30	1	Y	8.2
3	1	80	1	1	0	1	Y	5.6
3	1	100	1	0	520	0	N	1.2
3	0	120	1	1	200	1	Y	7.4
3	0	140	0	0	1150	1	N	3.1
3	0	120	0	1	75	1	N	7.8
2	1	80	1	0	1200	1	Y	1
3	0	120	1	1	450	1	N	10.1
3	0	150	0	1	60	1	N	7.4
3	0	130	1	1	50	1	N	2.5
3	0	130	1	1	400	1	Y	6.1
2	0	126	0	0	1200	0	N	1.3
2	0	120	0	0	1400	0	N	1.2
2	0	120	0	1	400	0	N	1.1
3	0	140	0	1	200	1	N	12
2	0	120	0	0	2100	0	N	0.8
3	0	130	0	0	2200	1	N	2
3	0	116	1	0	1200	0	N	1.2
2	1	90	0	0	1000	0	N	0.7
3	0	110	0	0	2300	0	N	#NULL!
2	0	110	0	0	1500	1	N	1.2
2	0	110	0	0	1400	1	N	2.6
2	0	200	0	0	800	0	N	3.8
3	1	100	0	1	200	1	Y	8.7
3	1	100	0	1	150	0	N	8
3	1	100	0	1	200	0	N	7.4
1	0	110	0	0	1250	0	N	1
3	0	110	0	0	800	0	N	1.8
2	0	110	0	0	1500	0	N	1.1
3	1	100	1	1	400	0	N	8.3
2	0	110	0	0	1300	1	N	0.8
2	0	120	0	0	2600	0	N	1.4
1	1	100	1	0	1600	0	N	1.7
2	0	120	0	0	2100	0	N	0.8
3	0	130	0	1	400	0	N	2.9
3	0	140	1	1	80	1	N	13.3

3	0	120	0	1	50	1	N	7.9
2	0	140	0	1	50	0	N	9.1
3	0	130	0	1	100	1	N	8.9
2	0	140	1	1	120	0	N	4.2
2	0	110	0	0	500	1	N	8.7
3	1	100	0	0	1200	0	N	1
3	0	130	0	0	950	0	Y	3.2
2	0	130	0	0	1500	0	N	0.9
3	0	110	1	1	50	0	N	5.1
3	1	100	0	0	950	0	N	1.3
3	0	130	1	0	1200	0	N	2.2
3	0	140	1	1	200	0	Y	6.1
3	0	140	0	0	1200	1	N	1.1
3	1	90	1	0	1500	1	N	0.7
3	0	130	0	1	0	0	N	9.3
3	0	130	0	0	1200	0	N	0.7
3	0	130	0	1	50	1	Y	6.1
2	0	120	1	0	1500	0	N	0.8
3	0	120	0	0	2100	0	N	0.1
3	0	120	0	0	1500	1	N	0.8
3	1	90	0	0	1850	1	N	0.8
3	1	90	1	1	0	1	Y	3.3
3	0	130	0	1	220	1	Y	7.3
2	0	110	0	0	1200	0	N	3.2
2	0	110	0	0	1500	0	N	2.2
3	0	140	0	1	450	0	Y	4.8
3	0	130	0	0	1300	0	N	1
3	0	110	0	0	1250	1	N	1
3	1	90	0	0	1500	1	N	5.4
3	1	80	1	1	40	1	Y	11.2
3	0	120	0	0	1250	1	N	0.7
2	1	100	0	0	1100	0	N	1
2	1	90	0	0	1450	0	N	0.9
3	0	160	0	0	1100	0	N	0.9
1	0	110	1	0	1500	0	N	3.2
3	1	0	1	1	0	1	Y	0.8
3	0	130	0	1	400	1	N	3.7
3	0	120	0	1	250	1	N	8
3	0	130	0	1	0	1	N	3.9
3	0	140	0	1	300	0	N	1.2
3	1	100	1	1	10	1	Y	4.8
3	0	120	0	0	1200	1	N	11.8
3	1	100	0	0	1200	1	N	2.4
3	0	136	0	0	1350	1	N	0.7
2	0	130	1	1	25	0	N	11.7
3	1	90	1	0	1200	1	N	0.9
3	1	90	0	1	50	1	N	4.2
3	1	70	1	1	0	1	Y	10
3	1	80	0	1	0	1	Y	5.2
3	1	90	1	1	0	1	Y	4

3	1	100	0	1	150	1	Y	56
3	1	0	1	1	0	1	Y	9.6
3	0	110	0	0	1200	1	N	0.8
3	0	120	0	0	1500	1	N	0.9
3	1	86	0	0	2000	1	N	0.9
2	0	120	0	0	1200	0	N	0.8
3	1	90	0	0	1800	0	N	1.1
3	0	120	0	0	1500	1	N	3.1
3	1	80	0	1	50	1	Y	3.1
2	0	120	1	1	80	1	Y	7.1
3	1	0	1	1	0	1	Y	9.1
2	1	0	0	1	0	1	Y	1.3
2	0	130	0	1	20	1	N	8.9
2	1	0	0	1	0	1	Y	6.1
3	0	120	1	1	0	1	Y	12
2	0	130	0	1	120	1	N	3.2
3	1	86	0	1	50	1	Y	3.2
3	1	100	0	1	25	1	Y	#NULL!
3	1	96	0	1	50	1	Y	#NULL!
3	0	120	0	1	100	0	Y	#NULL!
3	0	140	0	1	100	1	Y	9.3
3	0	130	0	1	200	1	Y	5.6
3	1	100	1	1	40	1	Y	2.1
2	1	0	1	1	0	0	Y	2.3
2	1	0	0	1	0	1	Y	1.9
3	1	80	1	1	0	1	Y	1.3
3	1	90	1	1	470	1	Y	6.5
3	0	130	0	0	540	1	Y	1.9
3	1	100	0	1	350	1	Y	1.21
3	1	94	0	1	0	1	Y	0.7
3	1	100	0	0	850	1	Y	5.6
3	1	80	0	1	40	1	Y	15.2
3	1	100	1	0	850	1	Y	4.2
3	1	80	0	1	200	1	Y	1.7
3	1	100	1	1	0	0	N	10.5
3	0	120	1	0	1400	0	N	4.5
3	0	130	0	1	200	1	N	3.7
3	0	130	0	0	1200	0	N	3.6
3	0	130	0	1	120	1	N	6.6
3	0	140	0	1	0	1	N	15.9
3	0	140	0	0	1000	1	N	1.1
2	0	190	0	1	100	0	N	11.2
2	0	130	0	0	750	0	N	0.9
3	0	110	0	0	2500	1	N	1.1
3	1	0	0	1	0	0	N	#NULL!
2	1	100	0	1	50	1	Y	#NULL!
3	1	80	0	1	150	1	N	6.3
3	1	80	0	1	50	1	N	9.2
3	0	120	0	1	0	1	Y	5.6
1	1	100	1	1	0	1	Y	9.2

1	0	130	0	0	1250	0	N	1
1	0	120	0	0	1250	0	N	1.2
3	1	100	0	1	60	0	N	5.3
3	0	120	0	0	1800	1	N	0.9
3	0	150	0	1	340	1	Y	7.8
1	0	120	0	0	1200	0	N	0.6
3	0	110	0	0	840	0	N	1.3
3	1	100	1	0	550	0	Y	5.7
2	0	120	0	0	1300	1	N	0.7
2	0	130	1	1	0	0	Y	4.8
2	0	130	0	0	1500	1	Y	0.8
0	0	120	0	0	800	0	N	0.9
2	1	100	1	0	500	1	N	6.5
1	0	120	0	0	1050	0	N	2.3
3	0	120	0	1	150	1	N	9.6
1	0	110	0	0	1000	0	N	2.2
2	0	150	1	1	295	1	N	5.8
3	0	150	0	0	600	0	N	8.8
2	0	130	0	0	1100	0	N	1.7
1	1	100	0	0	650	0	N	3.3
2	0	130	0	0	850	1	N	11.1
1	0	130	0	0	1000	0	N	5.8
3	0	120	0	0	660	0	N	11.2
1	0	110	0	1	400	0	Y	12.4
3	0	110	0	0	850	0	Y	9.6
3	0	120	1	1	250	1	N	19.9
2	0	130	0	1	400	1	Y	9.6
1	0	130	0	1	300	1	N	9.8
2	0	140	0	1	200	1	N	9.6
2	0	120	0	0	1100	1	N	10.8
2	0	110	0	1	0	0	N	7.7
2	1	100	0	0	1000	1	Y	4.3
1	0	138	0	1	270	1	N	5
3	0	132	0	1	240	1	N	9.2
3	0	123	0	1	250	1	N	8.4
2	0	120	0	1	440	0	N	1.7
3	0	130	0	1	50	0	N	5.3
2	1	80	0	0	900	0	N	0.8
1	0	180	0	0	2800	1	N	4.9
1	0	110	0	0	4000	1	N	0.8
0	1	100	0	0	2250	0	N	1.2
1	0	110	0	0	900	0	N	0.8
2	0	130	0	1	150	0	N	12.2
3	0	140	0	1	0	1	N	11.7
2	0	110	0	0	1550	1	N	0.6
2	1	100	0	0	2400	0	N	5.9
3	1	100	1	0	1200	0	N	0.8
2	0	110	0	0	1500	0	N	1.7
3	1	80	0	1	0	1	N	9.4

platelet_a	Thromboca	hb_a	asv_vials	totalasv	bitecodene	resultcode	GEN_CAT	score_12
287000	0	10.7	3	30	N	N	1	3
97000	0	12.9	19	190	N	N	1	1
25000	1	12	3	30	N	N	0	0
24000	1	6.9	0	0	N	N	1	2
30000	1	13.8	31	310	N	N	0	0
70000	0	10.1	10	100	N	N	0	2
130000	0	14	31	310	N	N	0	5
8000	1	4.1	31	310	Y	N	1	8
91000	0	14.2	20	200	Y	N	0	3
293000	0	9.4	43	430	Y	N	1	3
188000	0	12.3	20	200	N	N	0	0
120000	0	9.6	8	80	N	N	0	1
66000	0	13.1	19	190	N	N	1	3
231000	0	12.8	80	800	N	N	0	0
300000	0	12.5	19	190	N	N	0	3
184000	0	14.7	19	190	Y	N	0	1
174000	0	14	19	190	Y	N	0	3
107000	0	18.4	19	190	Y	N	0	4
81000	0	17.6	19	190	Y	N	0	1
82000	0	10.9	79	790	Y	N	0	1
98000	0	7.9	32	320	Y	N	0	4
96000	0	13.6	19	190	Y	N	0	4
10000	1	15.3	19	190	N	N	0	3
300000	0	10.2	31	310	Y	N	0	4
42000	1	16.4	19	190	Y	N	0	6
#NULL!	#NULL!	#NULL!	#NULL!	#NULL!	N	N	0	3
72000	0	13.3	29	290	Y	N	0	3
364000	0	9.6	0	0	Y	N	0	4
30000	1	12.5	0	0	Y	N	0	10
45000	1	13.3	0	0	N	N	0	0
106000	0	13.1	19	190	N	N	0	0
174000	0	7.6	45	450	N	N	1	2
107000	0	8.7	19	190	N	N	1	5
363000	0	9.7	84	840	Y	N	1	3
200000	0	12.5	5	50	N	N	1	1
96000	0	11.4	19	190	N	N	1	4
180000	0	12.3	3	30	N	N	1	6
154000	0	13.7	3	30	Y	N	0	1
#NULL!	#NULL!	#NULL!	#NULL!	#NULL!	Y	N	0	3
209000	0	12.9	16	160	N	N	0	0
40000	1	5.2	19	190	Y	N	0	7
65000	0	10.7	60	600	Y	N	0	3
8000	1	5.5	31	310	Y	N	0	7
15000	1	10.2	19	190	Y	N	0	4
50000	1	12.6	43	430	Y	N	1	5
245000	0	13.9	38	380	N	N	0	0
51000	0	14.7	19	190	N	N	0	3
80000	0	10.7	43	430	N	N	1	3
272000	0	6	3	30	Y	N	1	3

75000	0	12.9	31	310	N	N	0	2
45000	1	8.9	46	460	Y	Y	0	7
20000	1	7.4	73	730	N	Y	0	6
2000	1	4.8	61	610	Y	Y	1	10
45000	1	11.5	61	610	N	Y	0	7
11000	1	8.1	43	430	N	Y	1	9
85000	0	9.5	43	430	N	Y	0	8
96000	0	11.2	51	510	Y	Y	0	8
55000	0	9.6	31	310	N	Y	0	8
207000	0	11.1	134	1340	N	Y	1	4
13000	1	5.3	39	390	N	Y	0	6
40000	1	10.2	50	500	Y	Y	1	11
8000	1	6.5	18	180	N	Y	0	8
26000	1	8.3	18	180	N	Y	1	9
26000	1	5.2	21	210	N	Y	1	9
25000	1	7.6	30	300	N	Y	1	11
23000	1	9.6	5	50	Y	N	1	5
90000	0	7.2	83	830	N	Y	0	8
43000	1	13.1	31	310	Y	N	0	4
31000	1	15.3	71	710	N	N	0	5
22000	1	6.8	44	440	N	N	0	8
42000	1	9.2	31	310	N	N	0	6
67000	0	13.2	51	510	Y	N	0	6
69000	0	8.6	42	420	Y	N	0	7
80000	0	6.9	30	300	N	N	0	8
165000	0	11.2	26	260	Y	N	0	1
63000	0	12.9	13	130	N	N	0	0
20000	1	11.9	10	100	Y	N	0	3
84000	0	11.6	13	130	N	N	0	5
194000	0	14.2	10	100	Y	N	0	1
200000	0	11.4	0	0	Y	N	0	4
175000	0	8.5	30	300	N	N	0	1
294000	0	14.7	7	70	Y	N	0	3
176000	0	15.9	66	660	N	N	0	0
184000	0	12.5	7	70	Y	N	0	4
149000	0	11.1	19	190	Y	N	0	4
128000	0	10.1	10	100	Y	N	0	1
7000	1	10.5	10	100	N	N	0	9
19000	1	14.6	9	90	Y	N	0	5
11100	1	15	17	170	Y	N	0	5
84000	0	14.7	20	200	Y	N	0	1
26000	1	15	20	200	N	N	0	0
250000	0	11.9	33	330	Y	N	1	2
13000	1	9.7	26	260	Y	N	0	6
190000	0	11.4	31	310	Y	N	1	5
96000	0	10.1	96	960	N	N	1	1
95000	0	8.6	31	310	N	N	1	4
172000	0	13.3	30	300	N	N	1	1
68000	0	11.5	28	280	N	N	1	3
137000	0	5.9	10	100	N	N	0	6

67000	0	13.1	30	300	Y	N	0	6
116000	0	16.9	10	100	N	N	0	2
27000	1	12.8	45	450	N	N	0	5
138000	0	6.7	31	310	N	N	1	4
59000	0	10.7	50	500	Y	N	0	4
174000	0	14	31	310	Y	N	0	3
12000	1	18.3	31	310	N	N	0	2
42000	1	14.8	62	620	N	N	0	0
173000	0	6.9	31	310	N	N	0	3
100000	0	13.5	5	50	N	N	0	2
276000	0	8.4	55	550	N	N	0	1
8000	1	5.5	62	620	N	N	0	5
130000	0	10.4	43	430	N	N	1	4
130000	0	7.5	43	430	N	N	0	6
158000	0	14.4	52	520	N	N	0	2
197000	0	10.1	30	300	Y	N	0	1
17000	1	16.4	31	310	N	N	0	7
110000	0	8.6	8	80	N	N	0	1
204000	0	13.2	31	310	N	N	0	0
150000	0	10.2	10	100	N	N	0	3
147000	0	13.6	8	80	N	N	0	5
15000	1	10	65	650	N	Y	0	10
42000	1	10.3	8	80	N	N	0	7
133000	0	12.2	34	340	N	N	0	0
40000	1	14.4	43	430	N	N	0	0
15000	1	15.1	31	310	N	N	1	5
34000	1	11.5	31	310	N	N	1	1
201000	0	14.9	21	210	Y	N	1	5
15000	1	10.5	45	450	Y	N	1	7
10000	1	7.5	7	70	N	Y	0	10
32000	1	11.2	8	80	Y	N	1	5
118000	0	11.7	31	310	N	N	1	3
15000	1	12.7	31	310	N	N	1	3
186000	0	17.8	14	140	N	N	0	0
5000	1	3.8	15	150	Y	N	0	2
10000	1	7	20	200	N	Y	0	8
15000	1	12.8	80	800	N	N	1	6
20000	1	12.2	40	400	N	N	0	5
25000	1	11.2	46	460	N	N	1	6
58000	0	12.2	10	100	N	N	1	3
22000	1	8.6	51	510	Y	N	0	11
61000	0	11.5	37	340	Y	N	1	5
329000	0	16.9	30	300	Y	N	0	6
256000	0	14.7	5	50	Y	N	0	4
21000	1	10	21	210	Y	N	0	4
63000	0	7.4	12	120	N	N	0	6
56000	0	11.2	30	300	N	Y	0	7
5000	1	4.3	62	620	N	Y	1	11
52000	0	10.1	31	310	Y	Y	1	11
75000	0	9.1	51	510	Y	Y	0	9

46000	1	10.1	40	400	Y	Y	1	11
12000	1	8.5	30	300	Y	Y	0	11
111000	0	10.8	34	340	N	N	1	4
157000	0	14.6	7	70	N	N	0	3
39000	1	14.8	10	100	N	N	1	6
227000	0	13.3	21	210	N	N	0	0
140000	0	14.3	31	310	N	N	0	2
62000	0	11.2	30	300	Y	Y	1	5
41000	1	11.2	21	210	Y	Y	1	9
12000	1	9.1	30	300	N	Y	0	8
10000	1	5.3	70	700	Y	Y	0	11
15000	1	12.1	70	700	Y	Y	0	8
14000	1	15	40	400	Y	Y	0	6
10000	1	14.3	40	400	Y	Y	1	11
5000	1	5.6	25	250	Y	Y	1	10
96000	0	12	31	310	N	Y	0	5
30000	1	11	31	310	Y	Y	1	11
45000	1	15.2	10	100	Y	Y	0	10
10000	1	11.2	10	100	Y	Y	1	9
21000	1	11.2	45	450	Y	Y	1	6
16000	1	11.2	25	250	Y	Y	1	9
26000	1	10.2	25	250	Y	Y	0	8
10000	1	5.6	31	310	Y	Y	0	11
42000	1	9.6	31	310	Y	Y	1	9
58000	1	10.2	50	500	Y	Y	0	10
5000	1	8.6	15	150	Y	Y	1	12
100000	0	8.6	13	130	N	Y	1	11
25000	1	10.2	8	80	Y	Y	1	7
120000	0	15.8	48	480	Y	Y	1	11
65000	0	13.7	18	180	N	Y	0	9
24000	1	13.4	31	310	N	Y	0	7
14000	1	10.2	52	520	N	Y	0	9
100000	0	9.4	43	430	Y	Y	0	9
26000	1	15	25	250	Y	Y	0	10
35000	1	8.1	15	150	Y	N	1	7
30000	1	5.3	32	240	Y	N	0	2
60000	0	12.1	27	270	N	N	1	6
13000	1	13.1	20	200	N	N	0	0
76000	0	12.1	22	220	N	N	1	6
69000	0	102	5	50	Y	N	0	6
110000	0	10.3	31	310	N	N	0	3
210000	0	12.2	20	200	Y	N	1	4
80000	0	10.2	34	340	N	N	0	0
98000	0	10.1	15	150	N	N	0	3
12000	1	11.5	40	400	Y	Y	0	5
50000	1	10.3	40	400	Y	Y	1	11
12000	1	16.3	31	310	N	Y	0	7
8000	1	12.1	41	410	Y	N	0	8
55000	0	12.2	44	440	Y	Y	1	9
20000	1	5.3	42	420	Y	Y	1	11

120000	0	12	11	110	N	N	0	0
150000	0	13	10	100	N	N	1	1
12000	1	10.1	15	150		N	1	6
256000	0	12.3	10	100		N	1	5
23000	1	11.2	54	540		N	1	7
152000	0	10.2	10	100		N	0	0
138000	0	18.8	13	130		N	0	1
13000	1	6.4	32	320		N	0	5
150000	0	10.5	31	310		N	0	4
143000	0	5.6	31	310		N	0	6
32000	1	16.9	#NULL!	350		N	0	6
116000	0	17.9	0	0		N	0	0
30000	1	8.9	91	800		N	0	7
95000	0	10.2	36	360		N	0	0
14000	1	12	39	390		N	0	5
29000	1	12	34	340		N	0	0
92000	0	9.6	43	430		N	1	7
98000	0	12.5	8	80	N	N	0	1
150000	0	10.3	19	190		N	0	0
13000	1	12.3	31	310		N	0	3
62000	0	15.1	25	350		N	0	3
13000	1	16	35	350		N	1	1
26000	1	12.3	47	460		N	0	0
24000	1	13.2	40	400		N	0	5
15000	1	15.6	38	380		N	0	2
20000	1	8.5	31	310		N	0	6
46000	1	19.2	52	520		N	1	8
22000	1	11.2	41	410		N	1	6
32000	1	13.6	22	220		N	1	6
6000	1	14.2	30	300		N	0	3
46000	1	15.2	24	240		N	1	3
8000	1	12.3	46	460		N	0	7
30000	1	10.2	8	80		N	0	5
15000	1	14	34	340		N	0	6
15000	1	13.6	8	80		N	1	7
222000	0	12.3	8	80		N	0	2
65000	0	11.2	44	440		N	0	3
154000	0	11.1	3	30		N	1	3
267000	0	10.2	43	430		N	1	4
131000	0	11.2	20	200		N	0	3
165000	0	10.3	31	310		N	0	2
110000	0	12.8	43	430		N	0	1
80000	0	12.4	43	430		N	0	2
70000	0	10.6	8	80		N	0	6
91000	0	14.2	43	430		N	0	4
51000	0	14.7	31	310		N	0	3
150000	0	8.2	31	310		N	0	3
98000	0	12.5	43	430		N	0	0
8000	1	15	43	430		N	0	7

Outcome_cat
0
0
0
0
0
0
0
0
0
0
0
0
0
0
0
0
0
0
0
0
0
0
0
0
0
0
0
0
0
0
0
0
0
0
0
0
0
0
0
0
0
0
0
0
0
0
0
0
0

0
1
1
1
1
1
1
1
1
1
1
1
1
1
1
1
0
1
0
0
0
0
0
0
0
0
0
0
0
0
0
0
0
0
0
0
0
0
0
0
0
0
0
0
0
0
0
0
0
0

0
0
0
0
0
0
0
0
0
0
0
0
0
0
0
0
0
0
0
0
0
1
0
0
0
0
0
0
0
1
0
0
0
0
0
1
0
0
0
0
0
0
0
0
0
0
1
1
1
1

1
1
0
0
0
0
0
1
1
1
1
1
1
1
1
1
1
1
1
1
1
1
1
1
1
1
1
1
1
1
1
1
1
1
0
0
0
0
0
0
0
0
0
0
1
1
1
0
1
1

0
0
0
0
0
0
0
0
0
0
0
0
0
0
0
0
0
0
0
0
0
0
0
0
0
0
0
0
0
0
0
0
0
0
0
0
0
0
0
0
0
0
0
0
0
0
0
0
0
