# Supplementary material for: A simple mortality risk prediction score for viper envenoming in India (VENOMS): A model development and validation study
Source: PLoS Negl Trop Dis. 2022 Feb 22;16(2):e0010183. doi: 10.1371/journal.pntd.0010183 (PMC8896694; doi:10.1371/journal.pntd.0010183)
Supplement: S2 Data — (PDF) [file pntd.0010183.s004.pdf]

Serial no	age	sex	occupation	hospital_stay	part_bitten	snake	referral
1	41	2	1	8	1	1	1
2	50	2	1	3	1	1	1
3	60	1	1	4	2	1	1
4	35	2	1	4	1	1	1
5	33	1	1	3	1	1	0
6	69	1	1	4	1	1	1
7	24	1	1	5	1	1	1
8	35	2	1	14	1	1	1
9	35	2	2	7	1	1	1
10	53	1	3	3	1	1	1
11	25	1	1	4	1	1	1
12	40	1	1	4	1	1	0
13	35	2	1	3	1	1	0
14	32	1	1	3	1	2	0
15	65	2	1	7	1	2	0
16	53	1	1	8	1	1	0
17	41	1	1	7	1	1	1
18	60	1	1	4	1	1	0
19	28	1	1	2	2	2	0
20	24	1	1	13	1	1	1
21	35	2	1	18	1	1	1
22	45	1	1	15	1	1	1
23	19	1	3	4	1	1	0
24	27	1	1	3	2	1	1
25	23	1	1	4	1	1	0
26	65	1	1	7	1	1	1
27	18	1	1	5	2	1	1
28	23	1	1	5	1	1	0
29	28	1	1	29	1	1	1
30	60	1	1	21	1	1	1
31	30	1	1	6	2	1	1
32	54	1	1	5	1	1	1
33	40	1	1	5	1	1	0
34	55	1	1	5	1	1	0
35	55	1	1	7	1	1	1
36	31	1	1	4	1	1	1
37	48	1	1	5	1	2	1
38	44	1	1	5	1	1	1
39	50	2	1	3	1	2	1
40	35	1	1	4	1	2	0
41	15	1	1	3	1	1	0
42	45	2	1	5	1	2	0
43	28	1	1	12	1	1	0
44	40	2	1	18	1	1	0
45	60	1	1	2	1	1	0
46	30	2	1	1	1	1	1

47	41	1	1	4	1	2	1
48	59	1	1	30	1	1	0
49	36	2	1	5	1	1	1
50	54	1	1	18	1	1	0
51	21	1	3	3	1	1	0
52	50	2	1	3	2	1	1
53	32	2	1	5	2	1	1
54	13	1	3	3	1	1	0
55	68	2	1	11	1	1	0
56	58	1	1	5	1	2	1
57	21	1	1	3	1	2	1
58	32	2	1	3	1	1	1
59	24	1	3	7	2	1	0
60	45	1	1	7	2	1	1
61	55	1	1	13	1	1	0
62	57	1	1	3	1	1	1
63	44	2	1	3	1	1	1
64	58	1	1	7	2	1	1
65	39	2	1	5	1	1	1
66	25	1	1	4	1	1	1
67	24	1	1	4	1	1	1
68	42	1	1	4	2	1	0
69	56	1	3	6	3	1	1
70	30	2	1	1	1	1	1
71	35	2	1	3	1	1	1
72	29	1	1	6	1	1	1
73	25	1	1	6	1	1	1
74	40	1	1	3	1	2	0
75	45	1	1	6	1	1	1
76	70	1	1	5	2	1	0
77	31	1	1	1	1	1	1
78	45	1	1	5	1	1	0
79	38	1	1	6	1	1	1
80	56	1	1	2	1	1	0
81	35	1	1	6	2	1	0
82	75	2	2	2	1	1	0
83	25	1	1	20	1	1	1
84	42	1	1	3	1	1	0
85	35	1	1	1	1	1	1
86	13	2	3	6	1	1	1
87	25	1	1	4	1	1	1
88	48	1	1	9	1	1	1
89	25	2	2	18	1	1	0
90	30	1	1	2	1	1	0
91	30	1	1	1	1	1	0
92	49	1	1	4	1	1	1
93	30	2	1	7	2	1	1

94	40	1	1	8	1	1	0
95	50	1	1	5	1	1	1
96	39	1	1	5	2	1	1
97	31	1	1	3	1	1	1
98	75	1	1	3	1	1	0
99	13	2	3	8	4	1	1
100	38	1	1	7	1	1	0
101	17	1	1	7	3	1	1
102	53	1	1	6	1	1	1
103	18	1	1	6	1	1	1
104	18	1	1	4	1	1	0
105	25	1	1	4	1	1	1
106	35	2	1	3	1	1	0
107	39	1	1	4	1	1	0
108	37	2	2	6	1	2	0
109	27	1	1	7	1	1	0
110	33	1	1	22	1	1	1
111	22	1	1	4	1	1	1
112	40	1	1	5	1	1	1
113	27	1	1	5	1	2	1
114	35	1	1	5	1	1	0
115	33	1	1	6	1	1	0
116	49	1	1	4	1	1	0
117	37	1	1	12	1	1	1
118	37	1	1	3	1	1	0
119	76	1	1	2	1	1	0
120	18	1	1	10	1	2	0
121	50	2	1	6	2	2	0
122	33	2	1	3	1	1	0
123	30	2	1	10	2	1	1
124	47	1	3	2	1	1	1
125	40	2	2	3	1	1	0
126	23	2	1	3	1	1	0
127	40	2	1	9	3	1	0
128	32	1	1	4	1	1	1
129	45	2	1	3	1	1	1
130	40	1	1	4	1	1	0
131	45	2	1	11	1	2	1
132	42	2	1	24	1	1	1
133	35	1	1	2	1	1	1
134	50	1	1	5	1	1	1
135	55	1	1	8	1	1	1
136	15	1	1	3	1	1	1
137	55	1	1	2	1	1	1
138	38	1	1	6	2	1	1
139	21	1	3	7	1	1	1
140	34	1	3	3	1	1	0

AM_ASV_BA	cellulitis_A	bleeding_AA	pulse_AA	SBP_AA	DBP_AA	SpO2_AA	RR_AA
100	1	0	98	84	56	98	16
100	1	0	96	130	82	91	20
0	1	0	82	124	80	96	16
80	1	0	88	120	80	96	16
0	0	0	86	110	72	98	16
80	1	0	88	70	50	94	18
200	1	0	100	150	90	98	12
80	1	0	82	110	80	98	18
180	1	1	96	130	90	98	16
0	1	0	96	124	80	98	14
130	1	1	78	170	100	98	18
0	1	0	86	130	80	98	14
0	1	0	84	160	100	96	17
0	1	0	86	110	70	99	14
0	1	1	68	110	60	95	16
0	1	0	86	130	80	98	14
80	1	0	104	100	70	98	16
0	1	0	70	110	70	98	14
0	1	0	78	116	70	98	16
100	1	0	106	100	64	96	20
0	1	0	112	110	70	94	16
80	1	1	106	80	60	98	18
0	0	0	70	110	70	98	16
80	1	0	80	110	70	96	16
0	1	0	94	160	90	98	16
0	1	1	76	90	60	98	20
0	1	0	102	140	100	96	14
0	1	0	80	100	60	94	16
200	1	0	92	130	90	98	16
0	1	1	84	80	60	98	16
100	1	0	50	146	100	98	16
0	1	0	88	130	80	96	18
0	1	1	72	96	60	96	16
0	1	1	120	92	60	94	16
0	1	0	70	110	70	97	18
0	1	1	86	100	70	98	16
0	1	1	108	160	100	98	18
0	1	0	80	116	80	98	16
80	1	0	98	130	80	98	16
0	1	0	104	110	70	98	16
0	1	0	66	90	60	98	20
0	1	0	76	200	130	98	15
0	1	0	86	110	70	98	16
0	1	0	92	130	80	98	16
0	1	1	89	110	68	96	16
160	1	0	62	90	60	84	28

0	1	0	104	120	80	96	16
0	1	0	82	160	90	95	24
100	1	0	120	90	60	96	16
0	1	0	80	110	70	99	20
0	1	0	106	110	70	98	14
80	1	0	78	110	70	95	35
200	1	0	96	102	80	99	16
0	1	0	102	104	70	99	16
0	1	0	56	150	100	98	18
80	1	1	80	110	70	98	20
200	1	1	136	90	60	98	24
80	1	1	88	100	70	96	16
0	1	0	98	110	70	98	18
0	1	0	68	150	70	98	14
0	1	0	110	100	70	98	16
80	1	0	120	120	80	93	21
0	1	0	102	130	80	98	16
80	1	1	116	110	80	98	16
0	1	0	90	100	60	97	16
0	1	0	94	120	80	97	16
0	1	0	102	100	70	97	16
0	1	0	80	110	70	98	16
0	1	0	62	110	80	98	18
0	1	1	148	60	0	0	28
0	1	1	138	70	50	98	18
80	1	1	89	110	70	99	18
80	1	0	96	130	80	97	15
0	0	0	100	100	70	98	16
0	1	0	122	150	90	97	18
0	1	0	92	160	90	99	18
0	1	0	120	70	50	90	22
0	1	0	128	94	60	89	30
0	1	0	89	140	80	96	16
0	1	0	110	150	90	97	16
0	1	0	114	110	70	95	16
0	1	0	80	110	70	98	16
0	1	0	96	106	70	99	16
0	1	0	82	126	80	99	15
100	1	1	96	100	70	98	16
0	1	0	134	110	70	99	18
80	1	0	80	100	70	98	22
0	1	1	84	110	70	98	16
0	1	0	126	70	50	94	18
0	1	0	82	100	66	97	16
0	1	0	100	100	70	98	16
120	1	0	86	120	80	97	16
0	1	0	84	110	74	98	16

0	1	0	90	150	90	97	24
100	1	0	98	110	70	98	15
80	1	0	90	120	70	96	16
0	1	0	118	100	70	96	16
0	1	0	90	110	90	99	16
0	1	0	120	90	60	98	16
0	1	0	118	160	100	98	17
100	1	0	100	100	60	98	16
0	0	0	85	130	90	98	16
0	1	0	76	120	90	98	24
0	1	0	80	110	70	98	16
100	1	0	76	120	70	98	17
0	1	0	96	126	80	99	16
0	0	0	100	110	60	96	18
0	1	0	68	110	70	99	18
0	1	0	98	110	70	98	18
100	1	0	80	130	80	96	16
0	1	0	88	112	70	98	16
0	1	0	96	112	80	98	18
80	1	0	98	130	80	98	16
0	1	0	80	80	60	97	18
0	1	0	86	112	70	98	16
0	1	0	110	100	60	99	16
100	1	0	70	120	80	96	15
0	1	0	64	120	80	98	16
0	1	1	126	100	70	90	18
0	1	1	100	100	70	98	16
0	1	0	80	100	60	96	16
0	1	0	90	110	70	98	16
0	1	0	110	110	70	96	18
0	0	0	116	160	80	90	17
0	0	0	76	100	70	98	18
0	0	0	86	100	70	98	14
0	1	0	90	70	50	99	18
0	1	0	82	130	80	99	16
100	1	0	90	90	60	97	16
0	1	0	92	126	80	95	15
100	1	0	92	90	60	89	18
0	1	0	94	140	90	98	16
80	1	0	90	100	70	96	18
200	1	0	86	110	80	98	16
100	1	0	90	60	0	0	20
0	1	0	88	120	80	98	17
0	1	0	106	90	60	95	20
80	1	0	96	100	60	99	16
100	1	0	126	90	60	96	24
0	1	0	68	110	70	98	16

DBBTA	DBBTASV	DBBTAR	creat_AA	hb_AA	pla_AA	AKI	RRT_req	sev_cellu
14.5	9	15	0.9	11.9	300000	0	0	1
10	2	11	0.9	10.5	450000	0	0	1
5	6.75	6.75	1	10.8	150000	0	0	1
7	5	10	1.7	12.2	129000	1	0	1
1.5	1.75	1.75	1.2	13	214000	0	0	0
3.5	1	7.5	1.1	13	250000	0	0	1
5	1	11	0.9	14.3	206000	0	0	1
4.5	0.5	6.5	3.9	10.8	150000	1	0	1
50	3	50	3.5	10.9	10000	1	0	1
4	4.5	4.5	0.6	10.9	246000	0	0	0
25.5	1.5	26	4	9.6	35000	1	1	1
7.5	12	12	2.5	10.1	174000	0	0	0
3.5	2	3.5	0.7	13.6	237000	0	0	1
1	2	2	1.1	13.2	125000	0	0	0
2.75	2.75	4	0.8	10.9	138000	0	0	1
3.5	5.5	5.5	1.3	15.6	73000	1	0	1
12	4	14	2.4	15.1	100000	1	0	1
5	9	9	1	13.2	183000	0	0	1
1.5	3	3	1.2	13.2	160000	0	0	0
10.5	1.5	11	7.1	9.7	67000	1	0	1
5.5	6	6	1.2	10.9	24000	1	1	1
6	4	8	1.5	7.1	19000	1	1	1
2	2.75	2.75	0.9	12.1	235000	0	0	0
5	3	7	0.8	13.5	245000	0	0	0
2	3	3	1.2	12.6	230000	0	0	0
2.5	3	3	1	15	200000	1	1	1
4	5.5	5.5	1.3	12.6	30000	1	1	1
1	3	3	0.8	13	12000	1	1	1
12	2	4	2.1	20.3	40000	1	1	1
3	4	4	1.2	9.5	276000	1	1	0
5	7	0.5	1.3	13.6	179000	1	0	1
11	5	12	1.9	12.8	126000	1	0	1
1.75	2.25	2.25	1.2	12.7	164000	1	0	1
1.5	3.5	3.5	1.3	9.7	150000	0	0	1
2	4	4	1.2	10	60000	0	0	1
1.5	3	3	1.2	12.9	171000	0	0	1
1.5	2.5	2.5	1.2	12.4	32000	1	0	1
3	5	5	1	11.4	86000	1	0	1
7.5	2	9	1.1	13	150000	1	0	1
0.75	1.75	1.75	1.2	16.9	200000	0	0	1
11	12	12	0.8	11.4	159000	0	0	0
7	8	8	3.2	8.4	49000	1	0	1
2.75	3.5	3.5	0.8	11.6	20000	1	1	1
2.25	4.25	4.25	0.7	12.1	120000	1	1	1
9	10	10	1.3	9.3	35000	1	1	1
14	2	15	2.3	9.1	63000	1	0	1

7	8	8	1.7	12.8	200000	1	0	1
11	14	14	2.5	10.3	18000	1	1	1
24	3	25	2.6	10.8	125000	1	1	1
1	2	2	1.2	9.7	90000	1	1	1
1	2	2	0.8	10.1	246000	0	0	0
17	5	19	1.4	10.7	105000	0	0	0
8	2	12	0.9	11.5	195000	0	0	1
0.5	2.5	2.5	1	14.1	200000	0	0	0
1	2	2	2	11.1	104000	1	1	0
3	1	6	1.5	9	50000	1	0	0
2.75	0.5	7	1.2	14.2	105000	0	0	0
3	0.5	4.5	0.7	10.8	181000	0	0	0
1	5	5	1.2	14.7	188000	1	0	0
5	7	7	1	12.2	94000	0	0	0
13	14.5	14.5	1.1	17.6	116000	1	1	0
6	1	8	1.5	12.7	105000	1	0	0
4.5	5.5	5.5	1.1	10	211000	0	0	0
11	2	12	1.9	10	135000	1	0	0
6	7.5	7.5	1.8	10.4	180000	1	0	0
5	8	8	1.3	106	130000	1	0	0
2	2.75	2.75	0.8	11.6	166000	0	0	0
2	2.5	2.5	0.9	16.7	75000	1	0	0
15	17	17	2.2	12.1	19000	1	0	0
9	9	9	2.3	10.1	100000	1	1	1
16	17.5	17.5	1.1	10.1	9000	1	1	1
9	2	10	1.4	13.1	126000	0	0	0
18	3	15	3.2	11	45000	1	1	0
3	11	11	1.2	10.8	104000	1	0	1
2.5	5	5	1	11.1	150000	1	0	1
1	1.5	1.5	1	6.3	124000	0	0	0
6	7	7	1.5	12.6	140000	1	1	1
1	1.5	1.5	1.2	12.6	120000	0	0	1
28	30	30	2.5	12.4	228000	1	0	0
4	5	5	0.7	13.1	150000	0	0	0
2	4	4	1.1	15.7	183000	1	0	1
1.5	2.5	2.5	0.9	13.2	200000	0	0	0
6	7	7	1.3	13.9	56000	1	1	1
1.5	2.5	2.5	1	11	94000	0	0	0
7	2	8.5	0.9	12	96000	1	1	1
1	1.75	1.75	0.5	9.2	182000	1	0	1
9	2	12	1.5	14.5	78000	0	0	0
4.25	5.5	5.5	0.67	14.3	144000	1	0	1
1	2	2	0.7	9.5	90000	0	0	1
4	5	5	1	14	227000	0	0	0
2.75	3.5	3.5	0.9	14.1	100000	1	1	1
9	2	11	0.9	13.7	120000	1	0	0
2	5	5	0.7	9.4	25000	1	1	1

1.5	2.5	2.5	0.8	13.2	89000	1	0	0
17.5	6	19	6	12.9	16000	1	1	1
9.5	1.5	10	1.1	13.3	87000	0	0	0
4	4.5	4.5	0.8	13.2	150000	0	0	0
3	4	4	1.2	11.6	70000	1	0	0
9.5	11	11	0.7	12.4	238000	0	0	0
0.5	1	1	0.9	12.4	106000	0	0	1
16	3	17	1.3	11.3	130000	0	0	0
1	8.5	8.5	0.7	12.8	60000	1	0	0
2.5	3	3	1.1	14.2	100000	1	0	0
4	6.5	6.5	0.8	13.4	200000	0	0	0
7.5	1	9	1.4	14.5	76000	1	1	1
3.5	4.5	4.5	0.9	12.7	266000	0	0	0
1.5	2.5	2.5	1.4	10.3	74000	1	0	0
3.5	5.25	5.25	0.8	11.2	58000	1	1	0
2	3.5	3.5	1.1	12.1	66000	1	0	1
13.5	3	14.5	2.9	13.8	130000	1	1	1
2.5	3.5	3.5	0.8	16.8	99000	0	0	0
2	3.5	3.5	1.1	16	132000	1	0	1
8	1	10	1.3	12.4	120000	1	0	0
2.5	3.5	3.5	1.04	12.3	103000	1	0	0
0.75	2.5	2.5	1.2	13.4	179000	0	0	1
8.75	10	10	1	9.9	150000	0	0	0
9	2	11	1.4	14.8	140000	1	0	1
1	2.5	2.5	0.8	13	200000	0	0	0
4	5	5	1.4	9.1	120000	1	1	1
1	1.5	1.5	1.3	16.8	60000	1	1	1
16	19.25	19.25	0.8	9.7	155000	1	0	1
2	3	3	0.9	13.6	140000	0	0	0
2.5	3	3	0.7	8.8	50000	1	0	1
17.25	18	18	4.35	10.3	153000	0	0	0
3	4	4	0.9	13.1	170000	0	0	0
2	2.5	2.5	0.6	13.6	250000	0	0	0
7	8	8	1.2	9.7	40000	0	0	1
2.5	4	4	0.9	13.5	164000	0	0	1
10	3	12	1.9	10.9	90000	1	1	1
3	4.5	4.5	1.2	14	150000	1	0	0
1.5	1	7	1.1	12.6	42000	0	0	0
5.5	6.5	6.5	1.5	9.4	129000	1	1	1
8	2	9	1.55	14.6	154000	1	1	0
15	1	16	1.25	13.5	105000	1	0	1
4	1	6	1.3	12.4	76000	1	0	1
1	5	5	0.8	9.3	266000	0	0	0
7	8	8	1.2	13.2	120000	1	1	1
3	1	6	1.1	13.1	190000	0	0	0
24	10	25.5	0.9	14.1	51000	0	0	0
8.75	11.5	11.5	1.2	11.1	200000	0	0	0

shock	ASV_total	ASV_after_ran	death	clinical_bleed
1	200	100	0	0
0	200	100	0	0
0	200	200	0	0
0	380	300	0	0
0	160	160	0	0
0	100	20	0	0
0	400	200	0	0
0	180	100	0	0
0	380	200	0	1
0	100	100	0	0
0	150	20	1	1
0	200	200	0	0
0	280	280	0	0
0	130	130	0	0
0	190	190	0	1
0	220	220	0	0
0	300	200	0	0
0	100	100	0	0
0	130	130	0	0
0	300	200	0	0
0	300	300	0	1
1	300	220	1	1
0	190	190	0	0
0	180	100	0	0
0	100	100	0	0
1	300	300	1	1
0	420	420	1	1
1	300	300	1	1
1	300	100	0	0
1	300	300	0	1
0	300	200	0	0
0	20	20	0	1
0	200	200	0	1
0	200	200	0	1
0	250	250	0	0
0	200	200	0	1
1	100	100	0	1
0	200	200	0	1
0	280	200	0	0
0	100	100	0	0
0	100	100	0	0
0	160	160	0	0
1	250	250	0	1
1	200	200	0	1
1	150	150	1	1
1	300	140	1	1

0	200	200	0	0
1	300	300	0	1
0	200	100	0	1
0	300	300	0	1
0	160	160	0	0
0	100	100	0	0
0	300	100	0	0
0	100	100	0	0
1	20	20	1	1
1	240	160	0	1
0	300	100	0	1
0	180	100	0	1
1	300	300	0	1
0	220	220	0	1
1	130	130	0	0
1	100	100	0	0
0	100	100	0	0
0	300	220	0	1
0	100	100	0	0
1	100	100	0	0
0	100	100	0	0
0	100	100	0	1
0	100	100	0	1
1	70	70	1	1
1	300	300	1	1
0	210	130	0	1
0	180	100	0	1
0	100	100	0	0
0	100	100	0	0
0	130	130	0	0
1	200	200	1	1
1	100	100	0	0
1	100	100	0	1
0	100	100	0	0
1	100	100	0	0
0	100	100	0	0
0	300	300	0	1
0	50	50	0	0
1	200	100	1	1
0	100	100	0	0
0	280	200	0	0
1	190	190	0	1
1	300	300	0	1
0	100	100	0	0
1	300	300	1	1
0	250	130	0	1
1	300	300	1	1

1	200	200	0	1
1	380	280	1	1
0	180	100	0	0
0	100	100	0	0
0	100	100	0	0
1	190	190	0	1
1	210	210	0	1
0	100	100	0	0
0	300	300	0	1
0	100	100	0	1
0	100	100	0	0
1	400	300	1	1
0	130	130	0	0
0	200	200	0	0
0	220	220	0	1
1	130	130	0	1
1	200	100	0	0
0	100	100	0	0
0	160	160	0	1
0	300	220	0	1
1	300	300	0	1
0	100	100	0	0
0	100	100	0	0
0	290	190	0	1
0	100	100	0	0
1	300	300	1	1
1	300	300	0	1
1	100	100	0	0
0	100	100	0	0
1	200	200	0	1
0	100	100	0	0
0	100	100	0	0
0	100	100	0	0
1	250	250	0	1
0	100	100	0	0
1	200	300	1	1
0	130	130	0	1
1	200	100	0	1
1	220	220	0	1
1	280	200	1	1
1	300	100	0	0
1	290	190	0	1
0	200	200	0	0
0	300	300	1	1
0	180	80	0	0
1	260	160	0	1
0	100	100	0	0
