# Supplementary material for: A simple mortality risk prediction score for viper envenoming in India (VENOMS): A model development and validation study
Source: PLoS Negl Trop Dis. 2022 Feb 22;16(2):e0010183. doi: 10.1371/journal.pntd.0010183 (PMC8896694; doi:10.1371/journal.pntd.0010183)
Supplement: Attachment — Submitted filename: Response to reviewers comments.docx [file pntd.0010183.s005.docx]

Response to reviewer’s comments

We thank the editors for considering our manuscript and providing valuable feedback on salient points. We are grateful to the reviewers for their critical comments and raising several important points which has now made the manuscript more robust.

| **Editorial/reviewers’ comments** | | **Authors’ Response** | **Changes in the manuscript (Page number/line no.)** |
| --- | --- | --- | --- |
| 1 | 1. A syndromic approach to identifying the offending snake may well have resulted in errors.  2. Clinical manifestations vary greatly across India, and this sample of bite victims is from a single site.  3. The scoring system has only been validated in a single centre and that this was the same centre as the derivation cohort. The score requires independent external validation in other settings.   The authors should acknowledge these major limitations in the methodology, and the discussion and conclusions should take these limitations into account (be less dogmatic). | We thank the editors for pointing out these limitations.  We have substantially revised the discussion to include these limitations early in the discussion and have modified the conclusion to reflect these points.  We have also modified the abstract and author summary. |  |
| 2 | Abstract line 26 and and intro line 124 - is there conclusive evidence to support that mortality is higher amongst viperid envenomings? I would have thought neurotoxic elapdis would have a higher case fatality rate? | We acknowledge that the overall mortality is possibly higher in Elapids in pre-hospital settings, especially with issues surrounding availability of ventilatory support.  We have removed this line from the abstract and modified it in text to reflect this.  However, several studies seem to suggest that mortality from viper envenoming is probably higher including those mentioned below.     1. Chaudhari TS, Patil TB, Paithankar MM, Gulhane RV, Patil MB. Predictors of mortality in patients of poisonous snake bite: Experience from a tertiary care hospital in Central India. *Int J Crit Illn Inj Sci*. 2014;4(2):101-107. doi:10.4103/2229-5151.134145 2. Alirol E, Sharma SK, Bawaskar HS, Kuch U, Chappuis F (2010) Snake Bite in South Asia: A Review. PLoS Negl Trop Dis 4(1): e603. https://doi.org/10.1371/journal.pntd.0000603 | Abstract Line 24 and 25  Introduction line 119 |
| 3 | Author summary line 55-57 - it is too early to suggest the score can be used to inform clinical practice. It has only been validated in a single centre, and that was the same centre as the derivation cohort. Suggest reword to, 'may become' or 'shows promise'. | Suggestion accepted and reworded as: The VENOMS score is a good predictor of the mortality in viper envenoming from southern India where Russell’s viper envenoming burden is high. The score may have potential applications in triaging patients and guiding management. | Abstract  Page 2  Lines 41-43 |
| 4 | Methods line 155 - specify if patients were enrolled consecutively for the derivation cohort? | Patients were consecutive. This is now mentioned in methods. | Methods  Page 7  Lines 139-141 |
| 5 | Methods Table 1 - Although this is based on previously published data, suggest moving this table to the results section. | As suggested the table has been moved to results section. | Results section  Page 11  Lines 233-34 |
| 6 | Methods table 2 - suggest removing this table. All of these covariates with corresponding ORs and 95% CIs should be listed in Table 3 of the results. | We have accepted this suggestion and have removed Table 2 and moved it to the supplementary appendix as Table S2.  We have not combined this with Table 3 it specifically shows step 5 of the backward elimination process in multivariable logistic regression.  A combined table as recommended has been shown in Supplementary appendix table S2 with ORs and 95%CI of all parameters (limited to the step in which the predictor variable has been eliminated in backward elimination, if not included in the final model)  We feel that this table is better suited in the appendix as it may hamper readability if included in the main text.  Table 3 (Currently table 2) has been modified as well. | Supplementary appendix Table S2  And Table 2 page 13  Line 262 |
| 7 | Choice of covariates - it seems unusual not to include biting species as a covariate as this seems like an important predictor of outcome. Was this not used due to missing data - in which case specify this in the paper? If there is too much missing data, in those cases where the species was known it would be helpful to present the mortality rate by species. This will show if, for example, those with known Russell's envenoming have a higher mortality than those with known Echis envenoming. | While we admit the strength of this suggestion, reliable species identification using morphology was available (using photograph or dead snake) in only 21% of derivation and 8% of validation cohort. Species identification by venom assay was not done as these are not routinely available. Only clinical syndromic diagnosis was applied.  Overall, 74% in the derivation and 79% in the validation cohort were identified as Russell’s viper. However, of the number of Russell’s viper bites in the remaining patients cannot be ascertained as they can only be syndromically diagnosed as “viper bites” with further species identification not possible. Hence including species as a covariate was not deemed viable.  Essentially the score applies largely to Russell’s viper bites. We have now acknowledged this in detail in the discussion section. | Discussion  Page 20  Lines 404-405 |
| 8 | Results Table 3 - Include all covariates that were entered into the logistic regression model. Include unadjusted (univariate) and adjusted (multivariate) ORs, 95% CIs and p values. | We have modified table2 and table 3 as mentioned above.  Supplementary table S2 is a combined table. | Table S2 |
| 9 | Results Table 3 - there is an error in the 'Hb 10' covariate. P value is <0.0001 yet 95% CI 0.84-6.10. If this covariate is a non-significant predictor I would suggest removing it from the scoring system. | We thank the reviewer for pointing out the error. The p value is now corrected in Table 3.  However, we have retained Hb as a predictor as the analysis plan was to keep all variables at Step 5 even if not significant. The reason for this was to retain a clinically significant predictor: i.e., bite to antivenom time in the score.  The score will have a poor applicability and acceptability among clinicians without this important parameter hence all predictors which were in step 5 have been retained for uniformity (Please see below point 10 for further explanation).  We recognise that the robustness of the model is compromised by including these predictors and have specifically stated this in the discussion. | Table 3  Table S2 |
| 10 | Results Table 4 - I would disagree with including 'gender' and 'bite to ASV' as predictors in the model. The confidence intervals cross 1 and therefore it is not clear if they are associated with a higher or lower risk of death. One would expect the model to be slightly more accurate without them? | We agree that taking into consideration only statistical significance: bite to ASV and gender are not significant, in fact the early version of the score (as presented in the Snakebite: Science to society meeting, Leiden, 2018) did not have these 2 parameters.  However, based on our clinical experience, the valuable expert feedback received at meeting we piloted the score with clinicians for useability (results not shown in the current manuscript), we are inclined to retain these as bite to ASV time remains a very important issue for clinicians in snakebite mortality. Most clinicians in the pilot survey opined that they were unlikely to use a score without this parameter.  Our centre is fortunately situated in an area with good ambulance connectivity and first responders such that most patients received ASV within hours however this is rarely the case. Removing these will severely limit the applicability of the score to any other setting as delay in antivenom delivery are a serious and common problem all over India. Hence, we have decided to retain the parameters despite the minor compromise in the accuracy of the score.  Indeed, the AUC after removing these parameters was very close: 0.946 (95% CI 0.917-0.975) compared to the current AUC of 0.948 (95% CI 0.920-0.981) | Results  Page 12  Line 256  Table S3 and S4 |
| 11 | Results line 306 - was multiple imputation necessary when 97.8% of data was available. Was missing data 'missing at random?' Unless data was not missing at random and this influenced the model, I would suggest not doing multiple imputation. I'm not a statistician so happy to be corrected if there is something I am missing | We are grateful for this pragmatic suggestion, and we have removed the multiple imputation from the study. | Page 14  276-281 |
| 12 | Results line 331 - request that the sensitivity, specificity, PPV and NPV is calculated for every score cut-off in the validation cohort and that this be included in an additional table. See Abouyannis et al 2011 Table 3 for an example (doi:10.1097/QAD.0b013e328349a414). PPV and NPV are particularly meaningful when applied to clinical decision making. | We remain indebted for this valuable suggestion. A table with sensitivity, specificity, PPV and NPV is calculated for every score cut-off in the validation cohort is now included as Table 5 in the manuscript. | Table 5  Page 17 |
| 13 | More emphasis should be placed on the fact that this scoring system has only been validated in a single centre and that this was the same centre as the derivation cohort. Highlight that the tool requires independent external validation in other settings with a high burden of Russell's viper envenoming. | This point has been highlighted specifically in the discussion section as suggested and mentioned above. | Page 18  Line 354  Page 20 lines 405-6  Lines 408  And conclusion |
| 14 | Appendix S3 is difficult to interpret as it shows the raw output from the logistic regression. | Appendix 3 (Imputation analysis) has been removed now as per reviewer suggestion. | Page 14  276-281 |
| 15 | Running title – This is misleading – needs to specify Indian Vipers. | We have modified the running head as “The VENOMS score for viper mortality in India” to make the implications clear | Page 1  Title page |
| 16 | Line 26 - Reported mortality is higher in viper envenoming compared to snakes – please correct. | We thank the reviewer for pointing out this error. We have removed this line now. |  |
| 17 | Author summary Line 57-58 It can also aid better decision making with respect to antivenom administration and other supportive care. The findings of this study do not support this statement, VENOMS tool was not validated as to whether patients should be administered antivenom/repeated antivenom | We agree to this suggestion. The author summary has been modified accordingly by removing this line and specifically mentioning the geographic area of the study as southern India. | Page 2  Author summary  Lines |
| 18 | Image quality of Figure 1 is poor. | Image 1 has been edited for clarity according to journal requirements | Fig 1 |
| 19 | Overall, I think the topic area and the findings of this paper are highly important and certainly worthy of acceptance once the above changes have been made. Particularly in India, where mortality rates from envenoming are high, there is a great need for validated scoring systems that clinicians can use to help inform their decisions. The major limitation is that this is single centre, and validated in the same centre, so generalisability is not confirmed. | We are grateful to the reviewer for the kind words and encouragement. We sincerely hope that the score will be of use to clinicians managing snakebites in Southern India and Sri Lanka with further prospective validation. |  |
| 20 | The authors should be congratulated for devising a severity scoring system using a systematic approach to development and validation for Viper envenoming in India. Some suggestions and concerns are outlined below. | We thank the reviewer for this encouraging comment. The suggestions and concerns are addressed below. |  |
| 21 | Syndromic approach to identifying snake may have resulted in errors in inclusion/exclusion | We acknowledge this limitation in the methods and discussion section as outlined above. | Discussion  Page 20  Lines 404-405 |
| 22 | Sampling was from one site only – we know that clinical manifestations vary greatly across India and Indian subcontinent. | We agree and acknowledge this in clearly in the discussion as per previous suggestions. | Discussion  Page 20  Lines 406-407 |
| 23 | Validation cohort selected limited patients with >200ml antivenom prior to presentation. This is likely to bias the patient selection to less severely envenomed patients (demonstrated in Table 1 with reduced complication rates between groups 1 and 2 and a reduced mortality rate for the validation group). | We agree and acknowledge this point specifically in the limitations | Discussion Page 21  Lines 425-26 |
| 24 | Authors need to define CLS and the time at which data points were collected – Was this at admission or at 24 hours? (Bearing in mind the median time to development of signs is 48 hours). | Clinical definition of CLS used has been included in the methods section under predictor variable selection. The time for definition in the derivation cohort was at 24h of admission | Methods  Page 8  Line 180 |
| 25 | Did the authors assess heart rate as a potential variable? I note this was not included in the list of 25 potential variables listed in the supplementary material. | We agree that heart rate is a physiologically important clinical parameter for assessment and might possibly play a role in early prognostic assessment as tachycardia may indicate blood loss CLS or venom induced myocardial dysfunction.  Both the derivation and validation cohort were cross sectional and heartrate data was widely variable for the same patient within day of admission, at 48 hours and at discharge in the derivation cohort with multiple varying values recorded in the same day but a single value at a common cut of point (say 48 hours after bite) was not available for all patients.  Also, heartrate data was not available for all in the validation cohort. Owing to these practical difficulties, we did not consider heart rate as a potential parameter.  However, we plan to include heart rate data in a planned prospective validation of the score. | -- |
| 26 | After selection of the variables for inclusion in the VENOMS score – I would like to see a comparison of mortality risk curves for data collected at different time points. E.g on admission, admission +12 hours, Admission +24 hours, Admission + 36 hours, Admission + 48 hours. If the time points mortality risk scores are not statistically significant for each time point, then it supports the statement that the score can be calculated at any point between presentation and 48 hours after. | We agree with the reviewer that this would be an ideal analysis, however the data for all variables at various different time points as suggested are not available as both the cohorts collected cross sectional data at specific time points (24 hours, 48 hours and day 7 or discharge in the derivation cohort and at enrollment in validation cohort).  We have modified this line to reflect this limitation  We welcome this suggestion for prospective score validation being planned. | -- |
| 27 | I believe the paragraph from Lines 438-449 should be communicated early in the discussion. | We have moved this section as suggested expanding on other potential ways in which this score can impact management | Page 19  Lines 364-67 |
| 28 | Please expand on how use of the VENOMS severity score may improve snakebite management? Are there examples in the literature? How have clinical severity scoring for other conditions improved care (such as CURB65)? | As suggested, we have expanded the section on ways in which this score can impact management using the examples of PSI Wells score and SSS early in the discussion | Page 19  Lines 364 -68 |
| 29 | As mentioned in comments, I believe the subjects outlined between Lines 396-412 are limitations of this study methodology and that practical prospective assessment of this assessment tool is needed from multiple study sites (external validation) to corroborate the study findings. | We have now specifically outlined these limitations in the discussion section and modified the discussion to reflect this | Discussion  Page 20  Lines 406-407 |
| 30 | All individual patient data sets should be made readily available to meet PLoS NTD data sharing statement. This is not presently provided. | We have now provided deidentified datasets of both cohorts as other files |  |
