# Supplementary material for: A simple mortality risk prediction score for viper envenoming in India (VENOMS): A model development and validation study
Source: PLoS Negl Trop Dis. 2022 Feb 22;16(2):e0010183. doi: 10.1371/journal.pntd.0010183 (PMC8896694; doi:10.1371/journal.pntd.0010183)
Supplement: Attachment — Submitted filename: Response to reviewers_PlosNTD_10012021.docx [file pntd.0010183.s006.docx]

Dear Dr Casewell and Dr de Silva,

We are grateful to the editorial team their cooperation and providing us with extra time during the pandemic to carry out the revision process.

We express our heartfelt gratitude to the reviewers who have taken a keen personal interest in the process and providing excellent feedback which we feel has resulted in the manuscript being clearer and technically sound.

The minor revisions suggested have been addressed. Please find detailed responses to the comments below.

***Reviewer #1:*** *Table 5 - I suggest making it clear that each row refers to a score cut-off. Perhaps rename 1st column as “Venom score cut-off” and add ‘≥’ in front of the 0-12. It is also a bit confusing that for sens/spec/ppv/npv the accuracy using the cut-off is used, whereas for number of patient and number of deaths the number with that specific score is used. Perhaps change column 2 and 3 to the number of patients with that cut-off (i.e., the first row (score cut-off ≥0) would be 140 patients and 20 deaths. Second row 110 patients and 20 deaths). It may be possible to do this a different way - it just needs to be more clear and consistent for the reader.

Table 5 - I think there may be errors in the values in this table. For score cut-off of ≥2 the sensitivity is 97.5%. But no deaths are missed at this cut-off so sensitivity should be 100%? With a score cut-off of ≥3 the sensitivity is 95% (seems correct as 1 of 20 deaths missed). Score cut-off of ≥4, 90% sensitivity but this should be 95% as still only one death missed? I haven’t checked spec/PPV and NPV but I would suggest double checking all of the values in Table 5.*

**Response**: We thank Dr Abouyannis for this valuable suggestion and the table has been edited to reflect score cut-off ≥ a point (0,1 2 etc). The sensitivity, specificity PPV and NPV have been edited accordingly and values rechecked for errors as suggested. We are happy that table 5 has now become clearer and more user friendly.

***Reviewer #1:*** *Typo with full stop mid sentence line 406*

**Response**: This error has been rectified.

***Reviewer #1:*** *An excellent study that has adopted a data driven approach to developing a risk score for predicting mortality outcome of people with viper envenoming in India. With minor changes, as highlighted above, I would suggest this study be accepted.*

**Response:** We are grateful and heartened by the encouraging comments and hope that the score might be the right step to ultimately help prevent snakebite mortality in India.

***Reviewer #2:*** *The authors should be congratulated on the considerable improvement to the manuscript, particularly in reference to its potential applicability to clinical use. I look forward to seeing it validated in a multi-centre prospective study!*

**Response:** We thank the reviewer for valuable suggestions which have resulted in immensely improving the manuscript and look forward to sharing the results of prospective validation in the future.

***Reviewer #2:*** *Please double check all citations correspond to the relevant reference (for example citation 38 does not correspond to reference 38 - (a letter in reference to another article)) and ensure that the list of references are referenced in accordance with journal specifications.*

**Response:** All references have been recked and ref 38 has been corrected

Regards

Dr Maya Gopalakrishnan
